# Frailty phenotype as mediator between systemic inflammation and osteoporosis and fracture risks: A prospective study

**DOI:** 10.1002/jcsm.13447

**Published:** 2024-03-11

**Authors:** Dongsheng Di, Haolong Zhou, Zhangbo Cui, Jianli Zhang, Qian Liu, Tingting Yuan, Tingting Zhou, Xiao Luo, Danyang Ling, Qi Wang

**Affiliations:** ^1^ Key Laboratory of Environment and Health, Ministry of Education and Ministry of Environmental Protection, Department of Epidemiology and Biostatistics, School of Public Health, Tongji Medical College Huazhong University of Science and Technology Wuhan China

**Keywords:** fracture, frailty, mediation, osteoporosis, systemic inflammation

## Abstract

**Background:**

Systemic inflammation and frailty have been implicated in osteoporosis (OP) and fracture risks; however, existing evidence remains limited and inconclusive. This study aimed to assess the associations of systemic inflammation and frailty phenotype with incident OP and fracture and to evaluate the mediating role of frailty phenotype.

**Methods:**

The present study analysed data from the UK Biobank, a comprehensive and representative dataset encompassing over 500 000 individuals from the general population. Baseline peripheral blood cell counts were employed to calculate the systemic inflammation markers, including neutrophil‐to‐lymphocyte ratio (NLR), platelet‐to‐lymphocyte ratio (PLR) and systemic immune‐inflammation index (SII). Frailty phenotype was assessed using five criteria, defined as frail (≥3 items met), pre‐frail (1–2 items met) and non‐frail (0 items met). OP and fracture events were confirmed through participants' health‐related records. Multivariable linear and Cox regression models were utilized, along with mediation analysis.

**Results:**

Increased systemic inflammation was associated with increased risks of OP and fracture. The corresponding hazard ratios and 95% confidence intervals (CIs) for OP risk per standard deviation increase in the log‐transformed NLR, PLR and SII were 1.113 (1.093–1.132), 1.098 (1.079–1.118) and 1.092 (1.073–1.111), and for fracture risk, they were 1.066 (1.051–1.082), 1.059 (1.044–1.075) and 1.073 (1.058–1.089), respectively. Compared with the non‐frail individuals, the pre‐frail and frail ones showed an elevated OP risk by 21.2% (95% CI: 16.5–26.2%) and 111.0% (95% CI: 98.1–124.8%), respectively, and an elevated fracture risk by 6.1% (95% CI: 2.8–9.5%) and 38.2% (95% CI: 30.7–46.2%), respectively. The systemic inflammation level demonstrated a positive association with frailty, with *β* (95% CI) of 0.034 (0.031–0.037), 0.026 (0.023–0.029) and 0.008 (0.005–0.011) in response to per standard deviation increment in log‐transformed SII, NLR and PLR, respectively. The frailty phenotype mediated the association between systemic inflammation and OP/fracture risk. Subgroup and sensitivity analyses confirmed the robustness of these findings.

**Conclusions:**

Systemic inflammation and frailty phenotype are independently linked to increased risks of OP and fracture. The frailty phenotype partially mediates the association between systemic inflammation and osteoporotic traits. These results highlight the significance of interventions targeting systemic inflammation and frailty in OP and fracture prevention and management.

## Introduction

Osteoporosis (OP), characterized by diminished bone mineral density (BMD) and bone microstructure degradation, constitutes a substantial and escalating public health concern.^1^ Previous studies have indicated that the risk of OP affects approximately one third of females and one fifth of males aged 50 years and older, with a consistent rise in prevalence among the middle‐aged and elderly population.^2^, ^3^ The growing incidence of OP and the subsequent osteoporotic fractures^4^ have placed a significant burden on public health services, prompting global initiatives to discover more effective preventive measures for OP. Early detection and implementation of protective interventions for OP are essential health‐care strategies, underscoring the need for novel risk factors or biomarkers to evaluate OP risk effectively.

Systemic inflammation, stemming from the release of proinflammatory cytokines and chronic activation of the innate immune system, is intricately linked to the development of numerous chronic diseases.^5^ Although cumulative evidence has clarified the role of inflammatory dysregulation in bone metabolism,^6^, ^7^, ^8^, ^9^ inconsistencies attributable to single biomarkers,^10^ cross‐sectional study design^11^ and limited sample sizes^12^ exist in the findings. To address this issue comprehensively, a systemic inflammation evaluation utilizing combined inflammation markers, including neutrophil‐to‐lymphocyte ratio (NLR), platelet‐to‐lymphocyte ratio (PLR) and systemic immune‐inflammation index (SII), is warranted to assess the relationship between inflammation and OP. In the meantime, large prospective studies are needed to provide high‐level evidence.

Frailty is defined as a dynamic clinical condition characterized by increased vulnerability due to aging‐related deterioration in psychological, physical and social functioning.^13^, ^14^ Primary aging arises from complex aging mechanisms influenced by underlying genetic, epigenetic and environmental factors,^15^ resulting in a vicious cycle characterized by progressive loss of muscle and bone mass and fat gain.^16^ Specifically, chronic inflammation is a crucial contributor to frailty, either directly or indirectly through other intermediate mechanisms.^17^ Substantial evidence from both cross‐sectional and longitudinal studies has established a significant positive association between elevated inflammation levels and the risk of frailty.^18^, ^19^, ^20^ For instance, a recent cohort study demonstrated that the incident risk of frailty in Chinese older adults increased with rising levels of NLR, PLR and SII.^20^ These findings provide robust evidence supporting inflammation as a potential driver of frailty development. Frailty and its accompanying mechanisms, such as inactivity, decreased strength and weight loss, are conditions known to accelerate the onset of OP^21^ and increase the likelihood of falls and fractures.^22^ However, the relationship between frailty and OP remains unclear. In a cross‐sectional study of 230 community‐dwelling older individuals, Ma et al. observed that self‐reported frailty could enhance the prediction of OP in addition to traditional risk factors.^23^ In contrast, Gerdhem et al.'s study of 993 75‐year‐old Swedish women revealed a null association between frailty and OP.^24^ Given these contradictory findings, exploring the relationship between frailty and OP/fracture in a prospective study emerges as an intriguing avenue of research.

In light of the foregoing evidence, we hypothesized that systemic inflammation affects OP and fracture risks. The effects of systemic inflammation on the skeleton may be partially mediated by frailty status. To address these issues, we used data from the UK Biobank, an extensive repository encompassing phenotypic and genotypic data from over 500 000 individuals within the general population.

## Methods

### Study design and participants

The data for this study were acquired from the UK Biobank (https://www.ukbiobank.ac.uk/) with the assigned application number 88159. Between 2006 and 2010, the UK Biobank gathered detailed information on various phenotypes and genotypes from nearly 500 000 individuals at 22 assessment centres across the United Kingdom. All participants provided written informed consent, and ethical approval was obtained from the North West Multi‐centre Research Ethics Committee (reference: 11/NW/0382).

Our primary objective was to investigate the association between systemic inflammation and the risk of OP while assessing how frailty mediates this association. Additionally, we conducted a secondary analysis with fracture as a result measure. To ensure the robustness of our research, we carefully selected and processed the data from the UK Biobank cohort. First, we excluded 10 634 participants with OP at baseline. Next, we excluded subjects without systemic inflammation markers and physical frailty data, creating systemic inflammation (*N* = 393 443) and frailty (*N* = 390 485) subcohorts. From these subcohorts, we further excluded those with a fracture history at baseline to explore the association of systemic inflammation, frailty and fractures. To examine the mediating role of frailty, we merged the two parts of the data. *Figure* *S1* provides a visual representation of the study design and participant selection process, illustrating the data refinement steps conducted in our investigation.

### Systemic inflammation measurements

We extracted data on baseline peripheral blood cell counts for neutrophils, lymphocytes and platelets. Neutrophils serve as vital markers of innate immunity, platelets may contribute to immune function and lymphocytes provide extensive information about adaptive immunity.^25^ Subsequently, we computed three measurements indicative of systemic inflammation status: the NLR, equalling neutrophil counts divided by lymphocyte counts; the PLR, equalling platelet counts divided by lymphocyte counts; and the SII, equalling the product of neutrophil counts and platelet counts divided by lymphocyte counts. Evidence from observational studies has demonstrated a significant association of these combined inflammation indicators with increased risks of dementia,^26^ diabetes,^27^ cardiovascular disease^28^ and mortality.^29^ The ability of these three combined inflammatory indicators to predict inflammatory status under various conditions has been demonstrated.^5^ The UK Biobank blood sample data quality assessment technique is available at https://biobank.ndph.ox.ac.uk/showcase/showcase/docs/biomarker_issues.pdf. The instrument reports 31 parameters, the details of which are available at https://biobank.ndph.ox.ac.uk/showcase/ukb/docs/haematology.pdf. Before conducting risk analyses, these three blood cell ratios were log transformed and standardized to ensure data consistency and comparability.

### Physical frailty assessment

We utilized the frailty phenotype to assess physical frailty, as it is a more clinically applicable and widely accepted epidemiological measure.^30^ The frailty phenotype encompasses five traits: weight loss, exhaustion, low physical activity, slow walking speed and low grip strength.^31^ To adapt the data for use in the UK Biobank, we referred to previous reports and made slight adjustments to the criteria,^32^ as outlined in *Table* *S1*. Frailty status was evaluated based on the sum of scores for all five frailty items, with a higher total score indicating a higher frailty level. Participants were categorized as frail (≥3 items met), pre‐frail (1–2 items met) or non‐frail (0 items met) accordingly.^32^

### Incident osteoporosis and fracture ascertainment

The incidence of OP and fracture was confirmed by health‐related records using the International Classification of Diseases, 10th revision codes as follows: OP (M80, M81 and M82) and fracture (M484, M485, M80, M843, M844, S12, S22, S32, S42, S52, S72, S82, T02, T08, T10 and T12). Health‐related data for the UK Biobank were sourced from self‐report, primary care, hospital admission and death certificates. The duration of follow‐up was determined by the participant's attendance at the assessment centre until the diagnosis of OP/fracture, death, loss to follow‐up or the end of follow‐up (19 July 2022), whichever occurred first.

### Assessment of covariates

The covariates in the analysis included age (continuous), sex (male and female), ethnicity (non‐White/European ethnicity vs. White/European ethnicity), physical activity level (low vs. moderate vs. high), body mass index (BMI) (<18.5 kg/m^2^ [underweight], 18.5 to <25.0 kg/m^2^ [normal weight], 25.0 to <30.0 kg/m^2^ [overweight] and ≥30.0 kg/m^2^ [obese]), annual household income (less than £18 000 vs. £18 000 to £30 999 vs. £31 000 to £51 999 vs. £52 000 to £100 000 vs. greater than £100 000), Townsend deprivation index (continuous), education level (no qualification vs. other qualification vs. college/university degree), smoking status (never vs. current vs. previous), drinking status (never vs. current vs. previous) and the use of nutrient and mineral supplementation (calcium, iron, selenium and glucosamine) (yes vs. no).

### Statistical analyses

#### Description of the characteristics

As applicable, the results were reported using mean and standard deviation (SD), median and interquartile range, or numbers and percentages. Baseline characteristics were compared among frailty phenotype categories using appropriate statistical tests, including analysis of variance (ANOVA), Kruskal–Wallis rank sum or *χ* ^2^ tests.

#### Association of systemic inflammation, frailty and osteoporosis/fracture risk

Hazard ratios (HRs) and their corresponding 95% confidence intervals (CIs) for the association of systemic inflammation, frailty and OP/fracture risk were estimated using a Cox proportional hazards model. In addition, beta coefficients (*β*) and 95% CIs for changes in frailty scores per SD increment in systemic inflammation markers were estimated using linear regression models. Moreover, stratification analyses were performed based on sex (male or female) and age group (middle adulthood < 60 years or later adulthood ≥ 60 years).^33^

To assess the multiplicative interactions between systemic inflammation markers and frailty and subgroup variables (sex and age), we included a product term of them in the model. As the terms in the interaction had more than two levels, we used the car::Anova() function to get the *P*_interaction_ from the multiple degrees of freedom test.

#### Mediation analysis

Causal mediation analyses were also conducted based on Cox proportional hazards models to explore the potential mediating role of frailty in associations between systemic inflammation and OP/fracture risk. The mediation proportion of frailty in the total effect of systemic inflammation on OP/fracture was estimated. Details of this method were described previously.^34^

#### Sensitivity analyses

Sensitivity analyses were performed to evaluate the robustness of the main findings. First, to address potential reverse causality, we repeated the main analyses after excluding those who suffered from incident OP/fracture in the first 2‐year follow‐up. Second, we included information on the regular use of the anti‐inflammatory drug aspirin as an additional covariate. Third, we added C‐reactive protein (CRP), an acute‐phase inflammation biomarker, as a covariate in the models. Fourth, to minimize the impact of poor health on frailty status, we performed the main analyses again after excluding participants with poor self‐rated health status at baseline.

## Results

### Participant characteristics

After excluding participants with prevalent OP at baseline and those without systemic inflammation markers and physical frailty data, we obtained the systemic inflammation (*N* = 393 443) and frailty (*N* = 390 485) subcohorts. The baseline characteristics of the two subcohorts are presented in *Table* *1*.

**Table 1:** Characteristics of participants at baseline

Characteristics	Systemic inflammation subcohort (*N* = 393 443)	Frailty phenotype subcohort (*N* = 390 485)			*P* value^a^
		Non‐frail (*N* = 142 960)	Pre‐frail (*N* = 221 552)	Frail (*N* = 25 973)	
Incident OP cases (%)	12 722 (3.23)	3892 (2.72)	7070 (3.19)	1537 (5.92)	<0.001
Systemic inflammation markers, median (IQR)					
SII	528.08 (392.10, 714.00)				
NLR	2.14 (1.67, 2.77)				
PLR	132.50 (105.68, 166.67)				
Age (years), mean ± SD	56.05 ± 8.08	56.49 ± 8.06	55.65 ± 8.09	56.64 ± 7.88	<0.001
Townsend deprivation index, mean ± SD	−1.37 ± 3.04	−1.69 ± 2.87	−1.35 ± 3.03	−0.25 ± 3.42	<0.001
Female (%)	203 686 (51.77)	65 410 (45.75)	121 053 (54.64)	16 485 (63.47)	<0.001
BMI (%)					<0.001
Normal	124 184 (31.56)	56 630 (39.61)	63 620 (28.72)	3190 (12.28)	
Underweight	1839 (0.47)	805 (0.56)	934 (0.42)	83 (0.32)	
Overweight	168 952 (42.94)	61 597 (43.09)	96 985 (43.78)	9119 (35.11)	
Obese	98 468 (25.03)	23 928 (16.74)	60 013 (27.09)	13 581 (52.29)	
Ethnicity (%)					<0.001
Non‐White/European ethnicity	17 993 (4.57)	4555 (3.19)	10 524 (4.75)	2166 (8.34)	
White/European ethnicity	375 450 (95.43)	138 405 (96.81)	211 028 (95.25)	23 807 (91.66)	
Physical activity level (%)					<0.001
Low	62 023 (18.76)	14 909 (12.06)	38 271 (20.45)	8485 (41.77)	
Moderate	135 353 (40.95)	48 056 (38.87)	80 129 (42.81)	7648 (37.65)	
High	133 192 (40.29)	60 682 (49.08)	68 783 (36.75)	4180 (20.58)	
Annual household income (%)					<0.001
Less than £18 000	86 884 (22.08)	25 416 (17.78)	47 761 (21.56)	10 789 (41.54)	
£18 000 to £30 999	99 678 (25.33)	36 739 (25.70)	55 295 (24.96)	6528 (25.13)	
£31 000 to £51 999	103 720 (26.36)	39 666 (27.75)	58 866 (26.57)	5158 (19.86)	
£52 000 to £100 000	81 423 (20.69)	31 890 (22.31)	47 361 (21.38)	2939 (11.32)	
Greater than £100 000	21 738 (5.53)	9249 (6.47)	12 269 (5.54)	559 (2.15)	
Education level (%)					<0.001
No qualification	57 497 (14.61)	17 683 (12.37)	30 931 (13.96)	6784 (26.12)	
Other qualification	197 341 (50.16)	70 732 (49.48)	112 058 (50.58)	13 117 (50.50)	
College/university degree	138 605 (35.23)	54 545 (38.15)	78 563 (35.46)	6072 (23.38)	
Nutrient and mineral supplementation (%)	40 172 (10.21)	13 902 (9.72)	23 128 (10.44)	3016 (11.61)	<0.001
Drinking (%)					<0.001
Never	14 480 (3.68)	4143 (2.90)	8071 (3.64)	1801 (6.93)	
Previous	13 255 (3.37)	3716 (2.60)	7229 (3.26)	1924 (7.41)	
Current	365 708 (92.95)	135 101 (94.50)	206 252 (93.09)	22 248 (85.66)	
Smoking (%)					<0.001
Never	214 210 (54.44)	79 469 (55.59)	121 061 (54.64)	12 385 (47.68)	
Previous	137 927 (35.06)	50 126 (35.06)	77 304 (34.89)	9564 (36.82)	
Current	41 306 (10.50)	13 365 (9.35)	23 187 (10.47)	4024 (15.49)	

Abbreviations: BMI, body mass index; IQR, interquartile range; NLR, neutrophil‐to‐lymphocyte ratio; OP, osteoporosis; PLR, platelet‐to‐lymphocyte ratio; SD, standard deviation; SII, systemic immune‐inflammation index.

^a^ Baseline characteristics were compared between frailty status using analysis of variance, Kruskal–Wallis rank sum and *χ* ^2^ tests, as appropriate.

During a median follow‐up period of 13.4 years, the systemic inflammation subcohort included 12 722 incident OP cases, while the frailty phenotype subcohort included 12 499 new OP cases. Further, after excluding individuals with prior fractures, 18 857 and 18 601 participants suffered new fractures in the two subcohorts, respectively.

At recruitment, participants in the systemic inflammation subcohort had a mean age of 56.05 years, with 51.77% being females (*Table* *1*). Most (95.43%) were White, 42.94% were overweight and 25.03% were obese. Besides, 92.95% were current drinkers, and 10.50% were current smokers. In the frailty phenotype subcohort, the frail individuals were more likely to be female, obese, slightly older, have low‐level physical activity, smoke and be less educated than non‐frail ones (*Table* *1*).

### Systemic inflammation and osteoporosis and fracture risks

**Figure 1:**
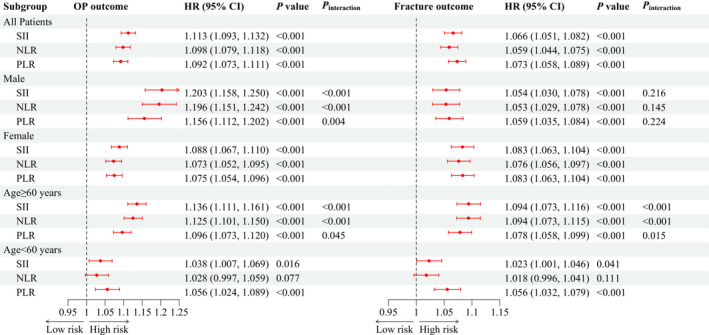
Hazard ratios (HRs) for incident osteoporosis (OP) and fracture in response to per standard deviation increment in systemic inflammation markers. CI, confidence interval; NLR, neutrophil‐to‐lymphocyte ratio; PLR, platelet‐to‐lymphocyte ratio; SII, systemic immune‐inflammation index. *P*_interaction_ indicates the test for interaction terms by subgroup variables (sex and age).

After adjusting for covariates, consistent positive associations were observed between SII, NLR and PLR with OP and fracture risks (*Figure* *1*). To be detailed, the HRs (95% CIs) for OP were 1.113 (1.093–1.132), 1.098 (1.079–1.118) and 1.092 (1.073–1.111) in response to per SD increment in log‐transformed SII, NLR and PLR, respectively. Similarly, the fracture risk increased by 6.6% (95% CI: 5.1–8.2%), 5.9% (95% CI: 4.4–7.5%) and 7.3% (95% CI: 5.8–8.9%) in response to per SD increment in log‐transformed SII, NLR and PLR, respectively.

The results of sex‐ and age‐stratification analyses maintained general consistency (*Figure* *1*). Sex acted as a modifier to the effect of systemic inflammation markers on OP risk (all *P*_interaction_ < 0.05), while not to the effects on fracture risk (all *P*_interaction_ > 0.05). The associations between systemic inflammation markers and OP/fracture risk all achieved significance in participants ≥60 years old (all *P* < 0.05), while the NLR and OP/fracture risk association was not significant in participants aged <60 years.

### Frailty and osteoporosis and fracture risks

**Figure 2:**
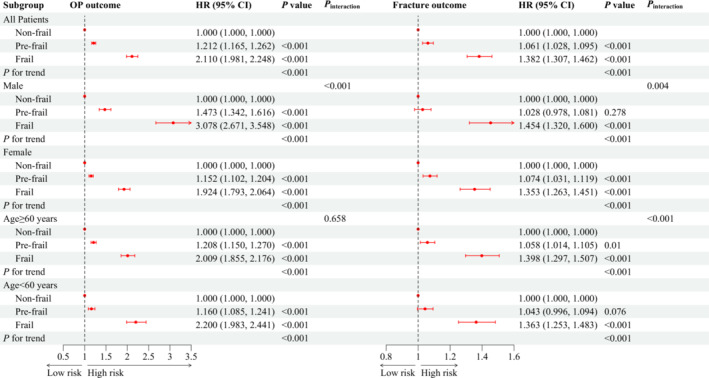
Association between frailty phenotype and incident osteoporosis (OP) and fracture risks. CI, confidence interval; HR, hazard ratio. *P*_interaction_ indicates the test for interaction terms by subgroup variables (sex and age).

*Figure* *2* illustrates the association between frailty status and OP/fracture risk while controlling for all the mentioned covariates. Pre‐frail and frail participants exhibited 21.2% (95% CI: 16.5–26.2%) and 111.0% (95% CI: 98.1–124.8%) higher risks of OP than did non‐frail individuals. Likewise, the risk of fracture increased by 6.1% (95% CI: 2.8–9.5%) and 38.2% (95% CI: 30.7–46.2%) among pre‐frail and frail individuals, respectively, compared with non‐frail individuals. Moreover, the association between frailty phenotype and the risk of incident OP/fracture exhibited a significant dose–response pattern (all *P*_trend_ < 0.001).

The findings from the age‐ and sex‐stratification analyses were largely consistent with the main analysis (*Figure* *2*). In the stratified analyses, a more substantial effect of frailty on the risk of OP/fracture was noted in males (*P*_interaction_ < 0.05), and a more pronounced effect of frailty on fracture risk was observed in participants over 60 years old (*P*_interaction_ < 0.001).

### Systemic inflammation and frailty

**Figure 3:**
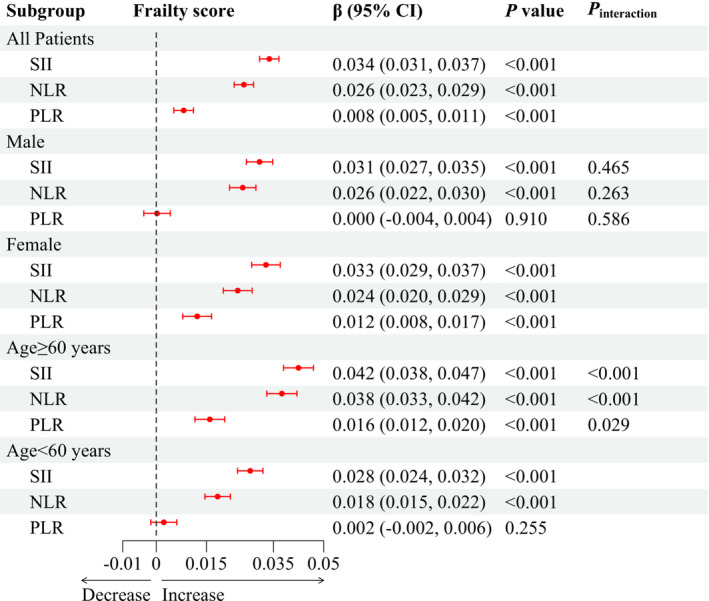
Association between systemic inflammation and frailty score. CI, confidence interval; NLR, neutrophil‐to‐lymphocyte ratio; PLR, platelet‐to‐lymphocyte ratio; SII, systemic immune‐inflammation index. *P*_interaction_ indicates the test for interaction terms by subgroup variables (sex and age).

After adjusting for covariates, an elevated systemic inflammation level demonstrated a positive correlation with an increased frailty score. Specifically, the *β* and their corresponding 95% CIs were as follows: *β* = 0.034 per SD of log‐transformed SII (95% CI: 0.031, 0.037), *β* = 0.026 per SD of log‐transformed NLR (95% CI: 0.023–0.029) and *β* = 0.008 per SD of log‐transformed PLR (95% CI: 0.005–0.011) (*Figure* *3*).

The results of stratification analyses by sex and age were generally consistent with the main analysis. Notably, age acted as a modifier to the association between systemic inflammation markers and frailty, with more pronounced effects of systemic inflammation markers on frailty scores in participants over 60 years old (all *P*_interaction_ < 0.05).

### Mediating role of frailty in systemic inflammation and osteoporosis/fracture association

Frailty had statistically significant but modest mediating effects on the associations between systemic inflammation markers (SII, NLR and PLR) and the risks of OP/fracture (*Table* *2*). Specifically, the indirect effects of frailty on SII–OP, NLR–OP and PLR–OP associations were 1.007 (95% CI: 1.007–1.008), 1.006 (95% CI: 1.005–1.007) and 1.002 (95% CI: 1.001–1.003), respectively, reflecting a minor increase in risk that, while statistically significant, was of limited magnitude. Similarly, the proportions mediated by frailty were under 5% in all inflammation marker–fracture risk relationships, although the large sample size allowed modest differences to reach significance.

**Table 2:** Mediating role of frailty in systemic inflammation and osteoporosis/fracture risk association

Outcome	Systemic inflammation markers	HR (95% CI)			Proportion of mediation
		Total effect	Direct effect	Indirect effect	
OP	SII	1.105 (1.085, 1.125)	1.096 (1.077, 1.116)	1.0074 (1.0065, 1.008)	7.39% (6.53%, 8.30%)
	NLR	1.090 (1.070, 1.110)	1.084 (1.064, 1.104)	1.006 (1.005, 1.007)	6.65% (5.75%, 7.59%)
	PLR	1.085 (1.066, 1.105)	1.083 (1.064, 1.103)	1.002 (1.001, 1.003)	2.21% (1.41%, 3.03%)
Fracture	SII	1.065 (1.049, 1.081)	1.062 (1.046, 1.078)	1.003 (1.002, 1.004)	4.68% (3.78%, 5.63%)
	NLR	1.057 (1.041, 1.073)	1.054 (1.039, 1.070)	1.002 (1.002, 1.003)	4.19% (3.35%, 5.09%)
	PLR	1.072 (1.056, 1.088)	1.071 (1.055, 1.087)	1.001 (1.000, 1.001)	1.03% (0.62%, 1.47%)

*Note*: Models were adjusted for age, sex, ethnicity, body mass index, annual household income, Townsend deprivation index, education level, drinking status, smoking status and nutrient and mineral supplementation. Abbreviations: CI, confidence interval; HR, hazard ratio; NLR, neutrophil‐to‐lymphocyte ratio; OP, osteoporosis; PLR, platelet‐to‐lymphocyte ratio; SII, systemic immune‐inflammation index.

### Results of sensitivity analyses

We performed several sensitivity analyses to validate the results obtained from the prospective analysis. Excluding participants who experienced incident OP or fracture during the original 2‐year follow‐up period, we repeated the data analysis and observed consistent associations between systemic inflammation, frailty phenotype and incident OP/fracture (*Table* *S2*). Furthermore, we further adjusted for CRP and regular aspirin use, and the results regarding the associations of systemic inflammation with OP and fractures remained robust (*Table* *S3*). Finally, the exclusion of participants with poor self‐rated health status did not alter the association between frailty phenotype and OP/fracture (*Table* *S4*).

## Discussion

This study constitutes a comprehensive prospective investigation examining the association between systemic inflammation, frailty phenotype and the risks of OP and fracture. Our findings demonstrated that elevated levels of systemic inflammation, as indicated by SII, NLR and PLR, were associated with increased risks of OP and fracture. Additionally, pre‐frail and frail individuals exhibited significantly higher risks of OP and fracture compared with non‐frail individuals. Furthermore, the mediation analysis revealed that frailty phenotype partially mediated the relationship between systemic inflammation markers and the incidence of OP and fractures.

Our findings regarding the relationship between systemic inflammation and OP/fracture are consistent with previous studies. For instance, Huang and Li found that an increasing NLR level was associated with an increased OP risk among Chinese postmenopausal women without diabetes.^6^ Additionally, a study by Fang et al. involving 238 Chinese postmenopausal women observed that a high SII level was a risk factor for OP.^12^ Besides, the SII level could discriminate against the risk of osteoporotic fracture in postmenopausal OP patients.^12^ These studies have validated the predictive value of the three inflammatory markers (NLR, PLR and SII) for OP and fracture risks. The underlying mechanism involves the disruption of bone homeostasis caused by the activation of the inflammatory microenvironment and a compromised immune system.^35^ Inflammatory cells residing in the bone marrow can trigger the release of cytokines and chemokines, resulting in an imbalance in bone function that favours osteoclast‐induced bone resorption.^36^, ^37^, ^38^ This immune‐inflammation imbalance ultimately results in osteopenia and weakened bone strength, contributing to the development of OP and fracture. Our findings highlight the clinical significance of systemic inflammation markers, such as NLR, PLR and SII, in providing valuable predictive information for OP and fracture risk.

Frailty is characterized by an age‐related decline in physiological reserve and function across multiple organ systems, resulting in a diminished capacity to withstand external stressors.^39^ Our findings suggested that the frailty phenotype is associated with the development of OP and fracture. Several potential mechanisms may contribute to the explanations. Sarcopenia, characterized by the loss of muscle mass and strength, is a critical component of physical frailty.^40^ Many studies have investigated the relationship between sarcopenia and OP. For instance, sarcopenia was strongly associated with OP among older Korean adults with chronic obstructive pulmonary disease.^S1^ Lima et al.^S2^ found that postmenopausal women with sarcopenia showed lower BMD values at all sites than those without, and those with severe sarcopenia had an OP risk 3.45 times greater than those without. Sarcopenia can lead to reduced mobility, falls and trauma, possibly precipitating fractures in individuals with OP.^S3^ Frail individuals are more prone to vitamin D deficiency and malnutrition,^S4^ which can exacerbate bone loss. Further, frailty and OP share common risk factors such as advanced age, low physical activity, weight loss and cognitive decline,^22^ suggesting shared biological pathways. Notably, OP and resultant fractures may exacerbate the frailty state, creating a vicious cycle in which the decline in physiological reserve and functional capacity is further aggravated. Hence, frailty and OP may interact in a complex, bidirectional manner.

It is widely accepted that plasma levels of inflammation indicated by markers (CRP, interleukin 6 and tumour necrosis factor α) are associated with frailty.^15^ Our findings from main and subgroup analyses have confirmed a significant positive association between systemic inflammation markers (NLR, PLR and SII) and frailty. Importantly, recent longitudinal evidence revealed that higher levels of NLR, PLR and SII were associated with increased incident risks of frailty, supporting inflammation as a contributor to frailty development.^20^ Although the exact mechanisms linking inflammation to frailty have not been fully elucidated, one plausible explanation is that inflammation is linked to decreased insulin‐like growth factor I synthesis and activity, which is crucial for muscle regeneration and maintaining muscle integrity.^S5^ The loss of skeletal muscle strength and mass is an integral aspect of frailty, which may be influenced by this inflammatory pathway. Further research is needed to fully elucidate the intricate mechanisms involved in the relationship between systemic inflammation and frailty.

In the UK Biobank cohort, frailty status was assessed using a Fried frailty phenotype model based on weight loss, exhaustion, walking pace, grip strength and physical activity level.^31^ Notably, grip strength is the only parameter obtained through objective determination. However, the absence of objective mobility measures such as gait speed in the UK Biobank dataset led us to rely on self‐reported walking pace as a proxy indicator of mobility.^32^ Nevertheless, it is important to acknowledge that the absence of such objective mobility measures may impact the accuracy and quantification of frailty assessments.

Although the mediation analysis suggested that frailty may play an intermediary role in the relationships between systemic inflammation markers (SII, NLR and PLR) and the risks of OP and fracture, the estimated mediation magnitudes were small. This finding suggested that the large sample size likely contributed to exaggerated statistical meanings of trivial differences.^S6^ Inflammation impacts bone health through complex mechanisms far beyond the narrow frailty pathway investigated here. Myriad biological, clinical and lifestyle factors are known to intertwine with inflammation and skeletal integrity.^S7^ ^–^ ^S9^ Isolating any singular mediator is an oversimplification of real‐world interactions. Thus, the statistically significant yet tiny mediating proportions should be interpreted prudently. While offering preliminary clues, our results cannot substantiate frailty as the key link bridging inflammation with compromised bone strength. Ultimately, confirming whether frailty truly intermediates inflammation‐triggered OP and fracture necessitates rigorous empirical intervention trials in future studies.

Our study has several strengths. First, this was a population‐wide prospective study with a substantial sample size and long follow‐up duration, providing ample statistical power to analyse the relationships between systemic inflammation and frailty and between frailty and the risk of OP/fracture. Second, we employed rigorous control measures for confounding factors, including socio‐economic status and lifestyle, and conducted comprehensive subgroup and sensitivity analyses to ensure the consistency and reproducibility of the results. However, several limitations warrant consideration. First, as an observational study, this study has a limited ability to establish causal relationships. Second, inflammatory markers and frailty phenotype were only assessed at baseline, lacking information on potential changes over time. Further research with repeated measurements during the follow‐up period is helpful to better understand the temporal association and potential impact on study outcomes. Third, our study focused solely on the UK Biobank cohort, and participants in this cohort may have distinct characteristics, such as being more health conscious and leading healthier lifestyles. Thus, future investigations involving populations with different characteristics and improved study designs are necessary to expand extrapolation. Fourth, the absence of objective mobility measures in the assessment of the frailty phenotype within the UK Biobank is a limitation. While self‐reported mobility proxies have demonstrated utility, the incorporation of quantified gait speed or other objective metrics could notably enhance the precision of frailty categorization. Future research should prioritize the inclusion of comprehensive, objective evaluations of physical functioning. Additionally, although the mediation analysis demonstrated statistically significant indirect effects of frailty, the estimated mediation magnitudes were quite small. The limited mediating role of frailty warrants cautious interpretation. Further studies are still needed to elucidate the pathways linking inflammation to bone health.

## Conclusions

In conclusion, our study highlights that systemic inflammation contributes to OP and fracture risks. Inflammation may participate in OP and fracture pathology by eliciting frailty. Our findings emphasize the importance of comprehensive prevention strategies for OP and fractures, which should include both anti‐inflammatory therapy and interventions to address frailty, particularly among high‐risk populations. Future research should focus on elucidating the underlying mechanisms and developing targeted interventions to break the inflammation–frailty–bone loss path, ultimately advancing OP and fracture prevention and management strategies.

## Funding

This study was supported by the National Natural Science Foundation of China (grant number 82273711) and the Funding for Scientific Research Projects from the Wuhan Municipal Health Commission (grant number WY22B06).

## Conflict of interest statement

The authors declare no potential conflicts of interest.

## Transparency

The lead author (the manuscript's guarantor) affirms that the manuscript is an honest, accurate and transparent account of the study being reported; that no important aspects of the study have been omitted; and that any discrepancies from the study as planned (and, if relevant, registered) have been explained.

## Supporting information


**Table S1.** Frailty phenotype items and designated scores used in the UK Biobank.
**Table S2.** Association between systemic inflammation, frailty and incident OP/fracture by excluding individuals who suffered from incident OP/fracture in the first 2‐year duration follow‐up.
**Table S3.** Association between systemic inflammation and incident OP and fracture by sensitivity analysis.
**Table S4.** Association between frailty phenotype and incident OP/fracture by excluding participants with poor self‐rated health status at baseline.
**Figure S1.** Flowchart of participant selection process. OP, osteoporosis.

**Data S1.** Supporting Information

## Data Availability

All UK Biobank information is available online on the webpage www.ukbiobank.co.uk. Data access is available through applications. This research was conducted using the application number 88159.
