# Supplementary material for: Frailty phenotype as mediator between systemic inflammation and osteoporosis and fracture risks: A prospective study
Source: J Cachexia Sarcopenia Muscle. 2024 Mar 11;15(3):897–906. doi: 10.1002/jcsm.13447 (PMC11154788; doi:10.1002/jcsm.13447)
Supplement: Supplementary file 1 — Table S1. Frailty phenotype items and designated scores used in the UK Biobank. Table S2. Association between systemic inflammation, frailty and incident OP/fracture by excluding individuals who suffered from incident OP/fracture in the first 2‐year duration follow‐up. Table S3. Association between systemic inflammation and incident OP and fracture by sensitivity analysis. Table S4. Association between frailty phenotype and incident OP/fracture by excluding participants with poor self‐rated health status at baseline. Figure S1. Flowchart of participant selection process. OP, osteoporosis. [file JCSM-15-897-s001.docx]

**Supplementary materials**

***Supplement table and figure***

Table S1. Frailty phenotype items and designated scores used in the UK Biobank.

Table S2. Association between systemic inflammation, frailty and incident OP/fracture by excluding individuals who suffered from incident OP/fracture in the first 2-year duration follow-up.

Table S3. Association between systemic inflammation and incident OP and fracture by sensitivity analysis.

Table S4. Association between frailty phenotype and incident OP/fracture by excluding participants with poor self-rated health status at baseline.

Figure S1. Flowchart of participant selection process. OP, osteoporosis.

Table S1. Frailty phenotype items and designated scores used in the UK Biobank.

| Number | Frailty phenotype items | Score |
| --- | --- | --- |
| 1 | Unintentional weight loss: Participants were asked “Compared with one year ago, has your weight changed?” | 1: “Yes, loss weight”; 0: Others |
| 2 | Exhaustion: Participants were asked “Over the past 2 weeks, how often have you felt tired or had little energy?” | 1: “More than half the days or nearly every day”; 0: Others |
| 3 | Weakness: Weakness was measured using grip strength with a Jamar J00105 hydraulic hand dynamometer (Lafayette Instrument). Participants were asked to complete a grip assessment for both hands once. The maximal value of the right and left hands was used. | 1: (1) Men: ≤29 kg for BMI ≤24 kg/m^2^; ≤30 kg for BMI 24.1-26 kg/m^2^; ≤30 kg for BMI 26.1-28 kg/m^2^; or ≤32 kg for BMI >28 kg/m^2^; (2) Women: ≤17 kg for BMI ≤23 kg/m^2^; ≤17.3 kg for BMI 23.1-26 kg/m^2^; ≤18 kg for BMI 26.1-29 kg/m^2^; or ≤21 kg for BMI >29 kg/m^2^; 0: Others |
| 4 | Slow gait speed: Participants were asked “How would you describe your usual walking pace?” | 1: “Slow pace”; 0: Others |
| 5 | Low physical activity: Participants were asked “In the last 4 weeks, did you spend any time doing light DIY^a^ activity, heavy DIY activity, or strenuous sports?” | 1: “None or light activity with a frequency of once per week or less”; 0: Others |

^a^DIY: do it yourself.

Table S2. Association between systemic inflammation, frailty and incident OP/fracture by excluding individuals who suffered from incident OP/fracture in the first 2-year duration follow-up^†^.

|  | OP | |  | Fracture | |
| --- | --- | --- | --- | --- | --- |
|  | HR (95% CI) | *P* value |  | HR (95% CI) | *P* value |
| Systemic inflammation markers |  |  |  |  |  |
| SII | 1.103 (1.083, 1.123) | <0.001 |  | 1.064 (1.047, 1.080) | <0.001 |
| NLR | 1.089 (1.069, 1.109) | <0.001 |  | 1.058 (1.041, 1.074) | <0.001 |
| PLR | 1.083 (1.064, 1.104) | <0.001 |  | 1.068 (1.051, 1.085) | <0.001 |
| Frailty phenotype |  |  |  |  |  |
| Non-frail | 1.000 (Reference) |  |  | 1.000 (Reference) |  |
| Pre-frail | 1.220 (1.170, 1.272) | <0.001 |  | 1.061 (1.025, 1.097) | <0.001 |
| Frail | 2.069 (1.937, 2.211) | <0.001 |  | 1.379 (1.299, 1.465) | <0.001 |
| *P* for trend | <0.001 |  |  | <0.001 |  |

^†^Models were adjusted for age, sex, ethnicity, physical activity level, BMI, annual household income, Townsend deprivation index, education level, drinking status, smoking status, nutrient and mineral supplementation.

Abbreviation: OP, osteoporosis; HR, hazard ratio; CI, confidence interval; NLR, neutrophil-to-lymphocyte ratio; PLR, platelet-to-lymphocyte ratio; SII, systemic immune-inflammation index.

Table S3. Association between systemic inflammation and incident OP and fracture by sensitivity analysis^†^.

| Sensitivity analysis | Systemic inflammation markers | OP | |  | Fracture | |
| --- | --- | --- | --- | --- | --- | --- |
|  |  | HR (95% CI) | *P* value |  | HR (95% CI) | *P* value |
| Adjusting CRP | SII | 1.100 (1.080, 1.121) | <0.001 |  | 1.058 (1.042, 1.074) | <0.001 |
|  | NLR | 1.086 (1.067, 1.106) | <0.001 |  | 1.051 (1.036, 1.067) | <0.001 |
|  | PLR | 1.086 (1.067, 1.106) | <0.001 |  | 1.067 (1.051, 1.083) | <0.001 |
| Adjusting regular aspirin use | SII | 1.112 (1.093, 1.132) | <0.001 |  | 1.065 (1.049, 1.080) | <0.001 |
|  | NLR | 1.098 (1.079, 1.118) | <0.001 |  | 1.058 (1.043, 1.073) | <0.001 |
|  | PLR | 1.093 (1.074, 1.113) | <0.001 |  | 1.074 (1.059, 1.090) | <0.001 |

^†^Primary models were adjusted for age, sex, ethnicity, physical activity level, BMI, annual household income, Townsend deprivation index, education level, drinking status, smoking status, nutrient and mineral supplementation.

Abbreviation: CRP, C-reactive protein; OP, osteoporosis; HR, hazard ratio; CI, confidence interval; NLR, neutrophil-to-lymphocyte ratio; PLR, platelet-to-lymphocyte ratio; SII, systemic immune-inflammation index.

Table S4. Association between frailty phenotype and incident OP/fracture by excluding participants with poor self-rated health status at baseline^†^.

| Frailty phenotype | OP | | Fracture | |
| --- | --- | --- | --- | --- |
|  | HR (95% CI) | *P* value | HR (95% CI) | *P* value |
| Non-frail | 1.000 (Reference) |  | 1.000 (Reference) |  |
| Pre-frail | 1.212 (1.164, 1.261) | <0.001 | 1.061 (1.028, 1.095) | <0.001 |
| Frail | 2.109 (1.979, 2.247) | <0.001 | 1.380 (1.304, 1.496) | <0.001 |
| *P* for trend | <0.001 |  | <0.001 |  |

^†^Models were adjusted for age, sex, ethnicity, BMI, annual household income, Townsend deprivation index, education level, drinking status, smoking status, and nutrient and mineral supplementation.

Abbreviation: OP, osteoporosis; HR, hazard ratio; CI, confidence interval.


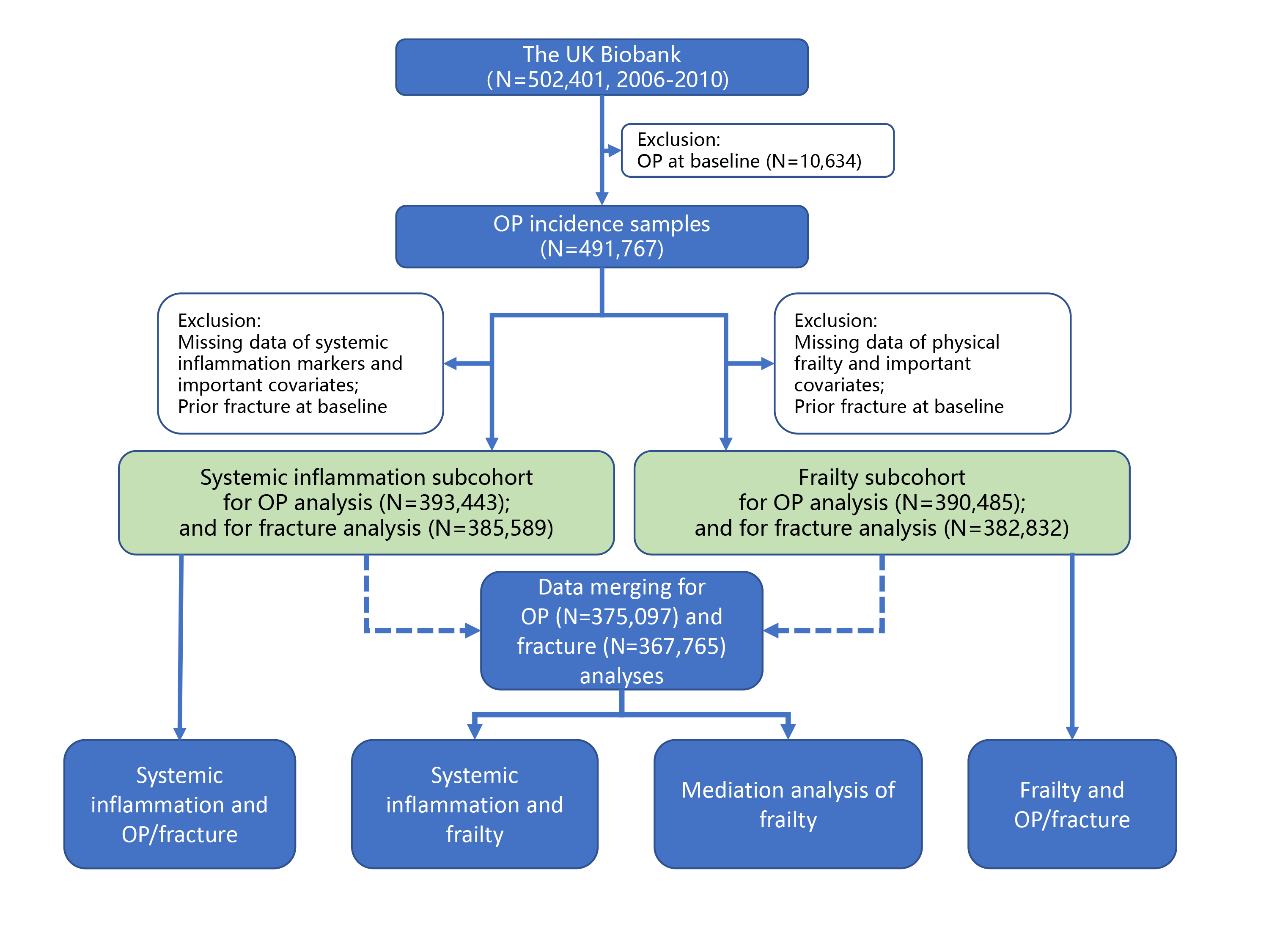


Figure S1. Flowchart of participant selection process. OP, osteoporosis.
