# Supplementary material for: Frailty phenotype as mediator between systemic inflammation and osteoporosis and fracture risks: A prospective study
Source: J Cachexia Sarcopenia Muscle. 2024 Mar 11;15(3):897–906. doi: 10.1002/jcsm.13447 (PMC11154788; doi:10.1002/jcsm.13447)
Supplement: Supplementary file 2 — Data S1. Supporting Information [file JCSM-15-897-s002.docx]

***Supplementary references***

S1. D. W. Lee and E. Y. Choi, *Sarcopenia as an Independent Risk Factor for Decreased BMD in COPD Patients: Korean National Health and Nutrition Examination Surveys IV and V (2008-2011).* PLoS One, 2016. **11**(10): p. e0164303.

S2. R. M. Lima, R. J. de Oliveira, R. Raposo, S. G. R. Neri, and A. B. Gadelha, *Stages of sarcopenia, bone mineral density, and the prevalence of osteoporosis in older women.* Arch Osteoporos, 2019. **14**(1): p. 38.

S3. A. J. Cruz-Jentoft and A. A. Sayer, *Sarcopenia.* Lancet, 2019. **393**(10191): p. 2636-2646.

S4. D. Marcos-Pérez, M. Sánchez-Flores, S. Proietti, S. Bonassi, S. Costa, J. P. Teixeira, et al., *Low Vitamin D Levels and Frailty Status in Older Adults: A Systematic Review and Meta-Analysis.* Nutrients, 2020. **12**(8).

S5. R. Sartori, V. Romanello, and M. Sandri, *Mechanisms of muscle atrophy and hypertrophy: implications in health and disease.* Nat Commun, 2021. **12**(1): p. 330.

S6. A. Gelman and J. Carlin, *Beyond Power Calculations: Assessing Type S (Sign) and Type M (Magnitude) Errors.* Perspect Psychol Sci, 2014. **9**(6): p. 641-51.

S7. J. J. Forsyth, *Menopause Osteoporosis and Bone Intervention Using Lifestyle Exercise: A Randomized Controlled Study.* J Midlife Health, 2023. **14**(2): p. 94-100.

S8. X. Lin, H. M. Xiao, H. M. Liu, W. Q. Lv, J. Greenbaum, R. Gong, et al., *Gut microbiota impacts bone via Bacteroides vulgatus-valeric acid-related pathways.* Nat Commun, 2023. **14**(1): p. 6853.

S9. T. Naidoo, L. L. Winchow, M. Tikly, and N. Govind, *Osteoporosis in Black South Africans With Rheumatoid Arthritis.* Cureus, 2023. **15**(10): p. e47743.
